# Microtubule dynamics in neuronal morphogenesis

**DOI:** 10.1098/rsob.130061

**Published:** 2013-07

**Authors:** Akira Sakakibara, Ryota Ando, Tamar Sapir, Teruyuki Tanaka

**Affiliations:** 1 Department of Anatomy and Cell Biology, Nagoya University Graduate School of Medicine, Nagoya 466-8550, Japan; 2 Department of Molecular Genetics, Weizmann Institute of Science, 76100 Rehovot, Israel; 3 Department of Developmental Medical Sciences, Graduate School of Medicine, University of Tokyo, Tokyo 113-0033, Japan

**Keywords:** microtubules, polarity, centrosome, axon, migration, neuron

## Abstract

Microtubules (MTs) are essential for neuronal morphogenesis in the developing brain. The MT cytoskeleton provides physical support to shape the fine structure of neuronal processes. MT-based motors play important roles in nucleokinesis, process formation and retraction. Regulation of MT stability downstream of extracellular cues is proposed to be critical for axonogenesis. Axons and dendrites exhibit different patterns of MT organization, underlying the divergent functions of these processes. Centrosomal positioning has drawn the attention of researchers because it is a major clue to understanding neuronal MT organization. In this review, we focus on how recent advances in live imaging have revealed the dynamics of MT organization and centrosome positioning during neural development.

## 2. Introduction

Neuronal migration and polarization are key activities in brain morphogenesis, and both rely on microtubule (MT) function [1–8]. MTs have intrinsic polarity based on the asymmetry of the αβ-tubulin heterodimer. MTs exhibit two distinct ends: a slow-growing minus end at which α-tubulin subunits are exposed, and a fast-growing plus end at which β-tubulin subunits are exposed [9,10]. MT network polarity within a cellular process affects not only its dynamic nature but also directed transport along MTs [3,6,9,10]. Formation of cytoplasmic MTs is initiated by binding of αβ-tubulin heterodimers to the γ-tubulin ring complex on the surface of an MT organizing centre such as the centrosome [9,10]. MT elongation through addition of tubulin heterodimers to the plus end forms a polarized cytoskeleton. Forming fibres undergo cycles of growth and shortening, a behaviour known as dynamic instability [11,12]. Neurons form large cellular protrusions such as leading processes, axons and dendrites, which function in neuronal migration and circuit formation. These processes contain an MT cytoskeleton, and dynamic changes in MTs underlie their extension and retraction [13–20]. Furthermore, the MT cytoskeleton is critical to maintain integrity of neuronal processes in the developing brain [21]. The highly polarized MT structure provides tracks for MT-based motors to enable directional movement of intracellular cargos within processes [22]. The minus-end-directed dynein motor complex plays a pivotal role in nucleokinesis in migrating neurons [23,24]. Kinesin super family proteins (KIFs), most of which are plus-end-directed motors, show multiple effects on MT dynamics and neuronal morphogenesis [22]. For example, kinesin-2 (KIF3) reportedly polarizes the Par3 complex leading to axon specification [25,26], whereas kinesin-1 (KIF5) promotes axon formation and elongation via transporting cargos such as membrane vesicles and the CRMP2–tubulin complex [27–29]. The mitotic MT-associated motor proteins kinesin-5 (Eg5, KIF11) and kinesin-12 (KIF15) negatively regulate short MT transport, limiting both axonal growth and neuronal migration [30–32]. Kinesin-6 (CHO1, MKLP1, KIF23) and kinesin-12 (HKLP2, KIF15) reportedly regulate MT organization in axons and dendrites [33]. Other kinesin family members, such as kinesin-8 (Kip3) and kinesin-13 (MCAK), are known to control dynamic instability by promoting MT catastrophe [34,35].

Defects in MT-related genes cause human diseases ranging from severe brain malformations to mental disorders [36–41]. Point mutations in genes encoding tubulin α- or β-subunits alter MT dynamics, and cause aberrant neurogenesis, migration and circuit formation [42–44]. The *LIS1* gene encodes a key regulator of the dynein complex [45–48]. Heterozygous mutations in *LIS1* or alterations in its normal dosage cause a range of developmental abnormalities, the most severe of which is lissencephaly (also known as smooth brain) [49,50]. Mutations in the X-linked *doublecortin* (*DCX*) gene, which encodes an unconventional MT-associated protein, underlie double cortex syndrome in humans [51–55]. The MT lattice-binding protein Tau is implicated in human intellectual disability and neurodegenerative tauopathies, among which are Alzheimer's disease, frontotemporal dementia and parkinsonism linked to chromosome 17 (FTDP-17) [56,57].

## 3. Microtubule function in the neuronal migration

Neuronal migration is central to proper neuronal alignment in the developing brain and requires orchestrated activity of cellular components, including cytoplasmic MTs [1,4,23]. During the development of cerebral cortex, three modes of migration have been described for excitatory neurons that are born in deep layers of brain, and then migrate radially towards the brain surface [2,4]. Those modes include somal translocation, multipolar migration and glial-guided radial migration or locomotion. Radial migration itself consists of three sequential steps: (i) leading process extension, (ii) nucleokinesis (i.e. nuclear translocation into the leading process) and (iii) retraction of the posterior end of the cell. In migrating neurons, the centrosome of migrating neurons is located in front of the nucleus (a phenomenon called N–C coupling). This configuration is considered crucial for nucleokinesis, as an N–C coupling is perturbed in migration-defective neurons [58–63].

### 3.1. Limitations of the dynein-based nucleokinesis model

**Figure 1.**
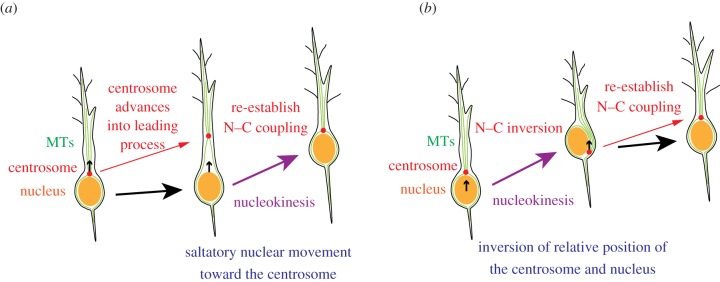
Potential function of MTs in nucleokinesis. (*a*) The dynein-dependent nucleokinesis model. In migrating neurons, the centrosome is positioned in front of the nucleus (N–C coupling). Cytoplasmic MTs derived from the centrosome surround the nucleus, and after the leading process elongates, the centrosome uncouples from the nucleus and advances into the leading process. Saltatory forward movement of the nucleus occurs using a minus-end-directed dynein motor along cytoplasmic MTs. (*b*) Occasionally, nucleokinesis occurs prior to centrosome movement, inverting the relative position of the centrosome and nucleus (N–C inversion). Centrosome positioning in front of the nucleus is then recaptured. This type of nucleokinesis cannot be explained by dynein-dependent pulling of the nucleus along cytoplasmic MTs. Small black arrows within the cells indicate the moving direction of centrosome or nucleus.

Based on the observation that the nucleus of migrating neurons is surrounded by a centrosome-derived MT cage [64], researchers have proposed a dynein-based nucleokinesis model [23]. According to this model, nucleokinesis consists of two steps: (i) centrosome uncoupling from the nucleus and advancement into a proximal ‘swelling’ in the leading process, and (ii) translocation of the nucleus to restore N–C coupling (figure 1*a*). The minus-end-directed motor, dynein, provides pull forces but cannot push the nucleus in this configuration. Importantly, centrosome-derived MTs orient their minus ends towards the centrosome and their plus ends towards the periphery. Thus, to explain this type of nucleokinesis by a dynein motor-based driving force, one must assume that forward movement of the centrosome always precedes nucleokinesis.

However, transient overtaking of the centrosome by a translocating nucleus has been observed in many types of migrating neurons (N–C inversion; figure 1*b*). Umeshima *et al.* [65] showed that nucleokinesis in migrating cerebellar granule cells is independent of centrosomal positioning. Distel *et al.*, report that the translocating nucleus is not consistently led by the centrosome in migrating zebrafish tegmental hindbrain neurons [66]. Furthermore, we observed similar inversion of relative positions of the leading centrosome and following nucleus in locomoting neocortical neurons in the developing mouse cerebrum [67]. In these cases, the migration cycle consists of two steps: (i) forward nuclear translocation into the leading process associated with overtaking of the centrosome, and (ii) re-establishment of the centrosome position in front of nucleus (figure 1*b*). Because the dynein-based nucleokinesis model cannot account for nuclear overtaking of the centrosome, a non-MT-based motor probably functions in this type of nucleokinesis.

### 3.2. Function of the actomyosin system in nucleokinesis

To explain mechanisms underlying the N–C inversion, the actomyosin system is an attractive candidate as a force generator [4,24]. Intracellular localization of actin filaments changes dynamically during nucleokinesis. Some investigators propose that a concentrated actomyosin system in front of a translocating nucleus pulls the nucleus [68], whereas others posit that actomyosin squeezes a translocating nucleus from the rear [69–71]. It is also plausible that cooperative force generation by dynein and actomyosin is used in nucleokinesis.

## 4. Microtubule regulation of neuronal polarization

Formation of functionally and structurally differentiated compartments such as axons and dendrites is a characteristic feature of neurons. Axons and dendrites emerge during a process known as neuronal polarization [26,72–74]. Each compartment contains distinct and highly organized MT-based networks whose roles in neuronal polarization have been investigated in various studies [2,3,5,6].

### 4.1. Function of microtubules in the spatial regulation of polarization signals

Axonogenesis, the extension of a single immature process and its differentiation into an axon, is the initial morphological event that defines neuronal polarization. Axonogenesis depends on extracellular cues that initiate signalling cascade leading to asymmetric distribution of components, including the polarity regulator, Par complex and phosphatidylinositol-triphosphate [25,26,75–77]. During the polarization process, MTs serve as tracks for translocation of polarity-regulating molecules. The plus-end-directed motor, kinesin-1, regulates spatially restricted specification of axons via its polarized transport activity [27,29]. CRMP-2, which binds to αβ-tubulin heterodimers, facilitates axon formation by transporting those heterodimers to the distal part of the axon via interaction with the kinesin-1 motor [28,78]. Local inactivation of GSK-3β by polarity signals reportedly regulates axonogenesis by controlling CRMP-2 affinity to tubulin [79]. The kinesin-3 motor protein GAKIN/KIF13B promotes axon specification by transport of phosphatidylinositol-triphosphate, which is also important for spatial restriction of axonogenesis signals [80]. These observations support the notion that plus-end-directed kinesin motors mobilize polarity signals in an emerging axon.

### 4.2. Regulation of microtubule stability in axon formation

Axons are typically thin, elongated processes that contain MTs with distinct organization and dynamics [3,5,6]. Altered MT stability underlies the dynamic nature of growth/retraction processes, as well as MT-based motor function. In axonogenesis, multiple protein kinases probably regulate MT remodelling in response to extracellular cues [62,63,81–85]. Kishi *et al.* [82] first found that SAD kinases phosphorylate Tau-1 S262, which is highly phosphorylated in dendrites but not in axons. Double knockout of genes encoding SAD-A and SAD-B kinases impairs axonogenesis, suggesting that initial axon formation is regulated by local inactivation of these kinases. LKB1 reportedly phosphorylates SAD kinases, and LKB1 loss of function promotes axonogenesis defects [83,84]. Others reported that local MT stabilization downstream of SAD kinases is important for axon specification in hippocampal neurons [86]. MARKs/Par-1, a kinase downstream of the aPKC/Par complex, phosphorylates DCX and reduces its MT-binding affinity [1,62,63,87,88]. The DLK–JNK pathway is implicated in axonogenesis via phosphorylation of the MT regulators DCX, MAP1B, MAP2 and SCG10/stathmin-2 [81,85,89,90], whereas others reported that local inactivation of stathmin/Op18 downstream of the DOCK7-Rac pathway regulates MT stabilization in axons [91]. Taken together, these observations suggest that regulation of MT stability by extracellular polarization signals is a key step in axonogenesis.

### 4.3. The role of centrosome translocation in axonogenesis

**Figure 2.**
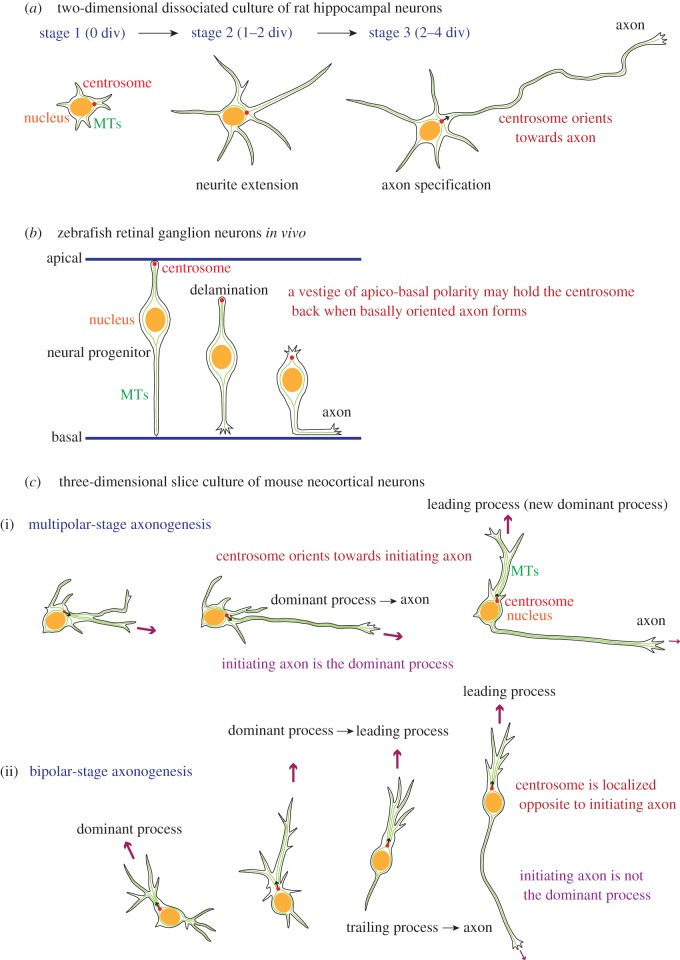
Centrosome positioning during neuronal polarization. (*a*) Rat hippocampal neurons cultured *in vitro* first extend multiple neurites (stage 2; 1–2 days *in vitro* (div)), and then one process differentiates into an axon (stage 3; 2–4 div). After axon specification, only the axon continues to extend, whereas other processes remain dynamic but short. In that case, the extending axon is likely to be the dominant process and attracts the centrosome (small black arrow). (*b*) In zebrafish retinal neural progenitors, the centrosome locates near the apical surface. After delamination, differentiated retinal ganglion neurons retain their bipolar shape along the apicobasal axis, and the centrosome stays on the apical side of the trailing process. The centrosome remains in the apical domain when an axon forms by extension of a basally oriented process, suggesting that a trace of apicobasal polarity may tether the centrosome. (*c*) Slice-cultured mouse neocortical excitatory neurons exhibit two modes of axonogenesis. (i) During multipolar (MP)-stage axonogenesis, an axon forms by extension of a dominant growing process (large purple arrows) targeted by the centrosome (small black arrows). (ii) Bipolar (BP)-stage axonogenesis is observed in neurons migrating radially towards the brain surface. An axon forms by extension of a thin trailing process from the rear of the cell, whereas the centrosome is located in front of the nucleus. In this case, the leading process (large purple arrow) rather than the initiating axon (small purple arrow) is likely to be the dominant process towards which the centrosome orients.

Centrosome translocation is observed during polarization of several types of neurons, but the significance of the dynamic nature of centrosomal positioning is unclear. Lefcort & Bentley [92] observed cytoskeletal organization of grasshopper pioneer neurons during axonogenesis and found that the axonal growth cone emerged preferentially at the opposite side of the cleavage point after the final cell division. The position of the centrosome after cell division corresponded to the axon emergence site, suggesting that centrosomal positioning determines axon orientation. Similar correlations of centrosome localization to the axon formation site have been observed during the early phase of axonogenesis in several types of neurons (figure 2*a*) [93–95]. These studies support the idea that centrosomal positioning plays an instructive role in determining axon orientation. However, there is a different conclusion in a recent manuscript from Dotti's laboratory reporting that localization of N-cadherin rather than centrosomal positioning specifies the first asymmetry in developing neurons [96].

By contrast, using zebrafish retinal ganglion cells, Zolessi *et al.* [97] observed a form of axonogenesis during which an axon emerges from the basal side of the cell body after a cell delaminates from the apical domain of the neuroepithelium/ventricular zone (figure 2*b*). In this case, the centrosome remained in the apical cytoplasm opposite the site of axon formation. Distel *et al.* [66] also observed the formation of thin axon fibres from the basal side of migrating tegmental hindbrain neurons in zebrafish. Forward extension of axon was initiated from the basal side of soma, whereas the centrosome was located apically in the trailing process.

Recently, we found that mouse neocortical excitatory neurons show two distinct modes of axonogenesis (figure 2*c*) [67]. The morphology of migrating neocortical neurons changes from multipolar to bipolar as cells migrate towards the brain surface [2,4]. Interestingly, we observed that centrosomes in these neurons tend to move towards the most dominant growing process. Multipolar migrating neurons form an axon by extending a dominant process towards which the centrosome orients. In bipolar locomoting neurons, the axon extends from the rear opposite the dominant leading process. During this mode of axonogenesis, the centrosome remained at the base of the leading process and did not target the initiating axon. Because we found that the centrosome in migrating neurons tends to move towards the most actively extending process, we concluded that centrosome positioning reflects relative protrusive activities of processes and that, in these cases, centrosome translocation during axonogenesis is likely to be a passive rather than an instructive event in orienting the axon.

## 5. Microtubule organization in axons and dendrites

**Figure 3.**
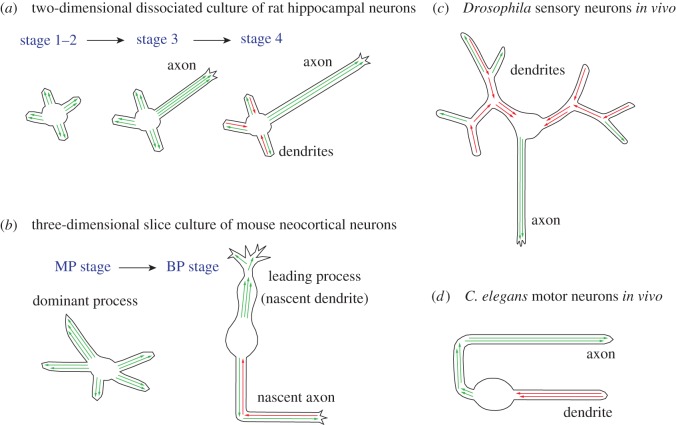
MT organization in axons and dendrites. (*a*) In the nascent process of *in vitro*-cultured rodent hippocampal neurons during polarization (stage 1–2), MT polarity is mostly plus-end-distal (green arrows). MTs are aligned in this manner in axons (stage 3–4). Minus-end-distal MTs (red arrows) increase in differentiating dendrites after stage 4. (*b*) Distal-oriented MT growth is predominantly observed in processes of MP-stage mouse neocortical neurons. The dominant process probably contains a greater number of MTs than do other processes and attracts the centrosome. Growing MTs are enriched in the leading process of BP-stage neurons, whereas bidirectional movements of MT plus ends are observed in the trailing axon. (*c*) *Drosophila* DA neurons exhibit highly branched sensory dendrites and a projecting axon. MTs are uniformly aligned in a plus-end-distal manner in the axon. MT polarity in dendrite shafts near the cell soma is mostly minus-end-distal, whereas short branches contain more plus-end-distal MTs. (*d*) In *C. elegans* motor neurons, polarity orientation of axonal MTs is mostly plus-end-distal. In dendrites, minus-end-distal alignment of MTs is predominant.

MT polarity within neurons affects not only process morphology but also motor protein-mediated transport, both of which have a profound effect on neuronal function [3,5–8]. Early electron microscopy studies using the *in situ* MT hook assay revealed that axonal MTs orient their plus ends towards the distal tip [8,98,99]. In analysing rat hippocampal neuron polarization in *in vitro* cultures, Baas *et al.* [100,101] further showed that MT polarity in neuronal processes dynamically changes as processes differentiate (figure 3*a*). In nascent neuronal processes, most MT plus ends are distally oriented. After dendritic processes mature, bidirectional MT alignment is observed, whereas MT polarity in the extended long axon remains mostly in a distal plus orientation [101]. By contrast, MT plus ends in the trailing axon of migrating granule cells in the developing cerebellum show mixed polarity, whereas MTs are uniformly aligned towards the growing tip of the leading process [102].

Progress in live imaging technology now allows analysis of the orientation of growing MT plus ends in living neurons. Stepanova *et al.* [103] first used EB3-EGFP to specifically label growing MTs plus ends in rodent hippocampal neurons cultured *in vitro*. They observed alignment of plus-end-distal MTs in axons and mixed-polarity MTs in dendrites is in agreement with results reported earlier using the MT hook assay [100]. Several groups have applied live imaging of MT plus ends to monitor MT polarity in neurons migrating *in vivo*. Tsai *et al.* [59] first reported that MT plus ends in the leading process of locomoting neurons point primarily towards the tip of the process. Recently, we further showed that the trailing axon of locomoting neurons in mouse embryonic cerebral slices contains mixed polarity MTs (figure 3*b*) [67]. These observations are consistent with observations of MT organization made in cerebellar granule cells *in vivo*, but not in hippocampal neurons *in vitro* [100,102]. Taken together, these results suggest that MTs show unique organization in two structural components of migrating neurons—namely, a major leading process in which organization is relatively uniform and a thin trailing axon in which MTs display mixed polarity.

MT organization patterns in *Drosophila* peripheral nervous system neurons differ from those seen in vertebrate neurons [7]. In *Drosophila* dendritic arborization (DA) neurons, MTs orient plus ends distally in the axon, whereas most growing MTs in dendrites orient plus ends proximally (figure 3*c*) [104]. In *Caenorhabditis elegans* sensory and motor neurons, most axonal MTs grow towards the distal tip, whereas retrograde MT growth towards the cell body predominates in dendrites (figure 3*d*) [105,106]. Thus, MT organization patterns in nematode neurons resemble those seen in insect neurons.

In *Drosophila* DA neurons, formation of uniformly oriented MTs in axons depends on dynein function [107], and morphogenesis of dendritic branches requires MT-based transport of Rab5-endosomes by dynein and kinesin [108]. Longer dendrites reportedly contain more retrogradely growing MT plus ends than do short branches, and MT organizing centres on Golgi outposts function in nucleation of MT polarity [109]. Overall, these findings suggest that an interplay between MTs, motor proteins and membrane organelles is critical for MT organization and neuronal process formation.

In mammalian hippocampal neurons, Golgi outposts are localized at branching points in dendrites [110], yet MT orientation nucleated from Golgi outposts has not been analysed. In slice-cultured mouse neocortical neurons, we observed generation of retrogradely growing MTs after retraction of the process tip [67], suggesting that MT severing and/or catastrophe near the tip of processes contributes to nucleation of mixed-polarity MTs [111].

Interestingly, cytoplasmic MTs can be organized independently of the centrosome in fly neurons [112,113]. It has also been demonstrated in rodent hippocampal neurons *in vitro* that once a nascent axon forms, a functional centrosome is dispensable for further axon extension [114]. These findings suggest that non-centrosomal MT nucleation functions to organize neuronal cytoplasmic MTs. In addition, transport of short MTs also participates in MT organization [33,115]. Taken together, multiple mechanisms probably regulate complex neuronal MT polarity. Further analyses of MT polarity in different neuronal subtypes and in diverse environments are needed to properly understand MT function in neuronal morphogenesis *in vivo*.

## 6. Concluding remarks

MTs, which are essential for cellular polarization and migration, exhibit unique structural and physical properties. Modulation of their structure is important for differentiation of immature processes into axons or dendrites. MTs serve as tracks for directed transport and as transducers of force generated by molecular motors, which control neuronal morphology and function. The importance of MT structure is highlighted by identification of mutations in tubulin genes and genes encoding MT-related proteins in patients showing brain defects or disorders. MT polarity within structural components of migrating neurons and polarized neurons has recently been described, yet how this organization is controlled requires further study. The centrosome, the main cellular MT organizing centre, displays dynamic behaviour during neuronal polarization and migration. Comparison of multiple cellular systems is currently promoting re-evaluation of its instructive function.

## References

[1] Reiner O Sapir T 2009 Polarity regulation in migrating neurons in the cortex. Mol. Neurobiol. 40, 1–14 (doi:10.1007/s12035-009-8065-0) 19330467 10.1007/s12035-009-8065-0

[2] Barnes AP Polleux F 2009 Establishment of axon-dendrite polarity in developing neurons. Annu. Rev. Neurosci. 32, 347–381 (doi:10.1146/annurev.neuro.31.060407.125536) 19400726 10.1146/annurev.neuro.31.060407.125536 PMC3170863

[3] Conde C Cáceres A 2009 Microtubule assembly, organization and dynamics in axons and dendrites. Nat. Rev. Neurosci. 10, 319–332 (doi:10.1038/nrn2631) 19377501 10.1038/nrn2631

[4] Marín O Valiente M Ge X Tsai LH 2010 Guiding neuronal cell migrations. Cold Spring Harb. Perspect. Biol. 2, a001834 (doi:10.1101/cshperspect.a001834) 20182622 10.1101/cshperspect.a001834 PMC2828271

[5] Kuijpers M Hoogenraad CC 2011 Centrosomes, microtubules and neuronal development. Mol. Cell. Neurosci. 48, 349–358 (doi:10.1016/j.mcn.2011.05.004) 21722732 10.1016/j.mcn.2011.05.004

[6] Stiess M Bradke F 2011 Neuronal polarization: the cytoskeleton leads the way. Dev. Neurobiol. 71, 430–444 (doi:10.1002/dneu.20849) 21557499 10.1002/dneu.20849

[7] Rolls MM 2011 Neuronal polarity in *Drosophila*: sorting out axons and dendrites. Dev. Neurobiol. 71, 419–429 (doi:10.1002/dneu.20836) 21557498 10.1002/dneu.20836 PMC4010183

[8] Baas PW Lin S 2011 Hooks and comets: the story of microtubule polarity orientation in the neuron. Dev. Neurobiol. 71, 403–418 (doi:10.1002/dneu.20818) 21557497 10.1002/dneu.20818 PMC3151545

[9] Jiang K Akhmanova A 2011 Microtubule tip-interacting proteins: a view from both ends. Curr. Opin. Cell Biol. 23, 94–101 (doi:10.1016/j.ceb.2010.08.008) 20817499 10.1016/j.ceb.2010.08.008

[10] de Forges H Bouissou A Perez F 2012 Interplay between microtubule dynamics and intracellular organization. Int. J. Biochem. Cell Biol. 44, 266–274 (doi:10.1016/j.biocel.2011.11.009) 22108200 10.1016/j.biocel.2011.11.009

[11] Mitchison T Kirschner M 1984 Dynamic instability of microtubule growth. Nature 312, 237–242 (doi:10.1038/312237a0) 6504138 10.1038/312237a0

[12] Bowne-Anderson H Zanic M Kauer M Howard J 2013 Microtubule dynamic instability: a new model with coupled GTP hydrolysis and multistep catastrophe. Bioessays 35, 452–461 (doi:10.1002/bies.201200131) 23532586 10.1002/bies.201200131 PMC3677417

[13] Lyser KM 1964 Early differentiation of motor neuroblasts in the chick embryo as studied by electron microscopy. I. General aspects. Dev. Biol. 10, 433–466 (doi:10.1016/0012-1606(64)90054-5) 14227358 10.1016/0012-1606(64)90054-5

[14] Lyser KM 1968 Early differentiation of motor neuroblasts in the chick embryo as studied by electron microscopy. II. Microtubules and neurofilaments. Dev. Biol. 17, 117–142 (doi:10.1016/0012-1606(68)90057-2) 5637101 10.1016/0012-1606(68)90057-2

[15] Tennyson VM 1965 Electron microscopic study of the developing neuroblast of the dorsal root ganglion of the rabbit embryo. J. Comp. Neurol. 124, 267–317 (doi:10.1002/cne.901240302) 5325036 10.1002/cne.901240302

[16] Tennyson VM 1970 The fine structure of the axon and growth cone of the dorsal root neuroblast of the rabbit embryo. J. Cell Biol. 44, 62–79 (doi:10.1083/jcb.44.1.62) 5409464 10.1083/jcb.44.1.62 PMC2107779

[17] Yamada KM Spooner BS Wessells NK 1970 Axon growth: roles of microfilaments and microtubules. Proc. Natl Acad. Sci. USA 66, 1206–1212 (doi:10.1073/pnas.66.4.1206) 5273449 10.1073/pnas.66.4.1206 PMC335807

[18] Yamada KM Spooner BS Wessells NK 1971 Ultrastructure and function of growth cones and axons of cultured nerve cells. J. Cell Biol. 49, 614–635 (doi:10.1083/jcb.49.3.614) 4326456 10.1083/jcb.49.3.614 PMC2108504

[19] Solomon F 1980 Neuroblastoma cells recapitulate their detailed neurite morphologies after reversible microtubule disassembly. Cell 21, 333–338 (doi:10.1016/0092-8674(80)90469-9) 7407915 10.1016/0092-8674(80)90469-9

[20] Suter DM Miller KE 2011 The emerging role of forces in axonal elongation. Prog. Neurobiol. 94, 91–101 (doi:10.1016/j.pneurobio.2011.04.002) 21527310 10.1016/j.pneurobio.2011.04.002 PMC3115633

[21] Sakakibara A Horwitz AF 2006 Mechanism of polarized protrusion formation on neuronal precursors migrating in the developing chicken cerebellum. J. Cell Sci. 119, 3583–3592 (doi:10.1242/jcs.03080) 16912080 10.1242/jcs.03080

[22] Hirokawa N Niwa S Tanaka Y 2010 Molecular motors in neurons: transport mechanisms and roles in brain function, development, and disease. Neuron 68, 610–638 (doi:10.1016/j.neuron.2010.09.039) 21092854 10.1016/j.neuron.2010.09.039

[23] Tsai LH Gleeson JG 2005 Nucleokinesis in neuronal migration. Neuron 46, 383–388 (doi:10.1016/j.neuron.2005.04.013) 15882636 10.1016/j.neuron.2005.04.013

[24] Vallee RB Seale GE Tsai J-W 2009 Emerging roles for myosin II and cytoplasmic dynein in migrating neurons and growth cones. Trends Cell Biol. 19, 347–355 (doi:10.1016/j.tcb.2009.03.009) 19524440 10.1016/j.tcb.2009.03.009 PMC2844727

[25] Nishimura T Kato K Yamaguchi T Fukata Y Ohno S Kaibuchi K 2004 Role of the PAR-3-KIF3 complex in the establishment of neuronal polarity. Nat. Cell Biol. 6, 328–334 (doi:10.1038/ncb1118) 15048131 10.1038/ncb1118

[26] Arimura N Kaibuchi K 2007 Neuronal polarity: from extracellular signals to intracellular mechanisms. Nat. Rev. Neurosci. 8, 194–205 (doi:10.1038/nrn2056) 17311006 10.1038/nrn2056

[27] Nakata T Hirokawa N 2003 Microtubules provide directional cues for polarized axonal transport through interaction with kinesin motor head. J. Cell Biol. 162, 1045–1055 (doi:10.1083/jcb.200302175) 12975348 10.1083/jcb.200302175 PMC2172855

[28] Kimura T Arimura N Fukata Y Watanabe H Iwamatsu A Kaibuchi K 2005 Tubulin and CRMP-2 complex is transported via kinesin-1. J. Neurochem. 93, 1371–1382 (doi:10.1111/j.1471-4159.2005.03063.x) 15935053 10.1111/j.1471-4159.2005.03063.x

[29] Jacobson C Schnapp B Banker GA 2006 A change in the selective translocation of the kinesin-1 motor domain marks the initial specification of the axon. Neuron 49, 797–804 (doi:10.1016/j.neuron.2006.02.005) 16543128 10.1016/j.neuron.2006.02.005

[30] Myers KA Baas PW 2007 Kinesin-5 regulates the growth of the axon by acting as a brake on its microtubule array. J. Cell Biol. 178, 1081–1091 (doi:10.1083/jcb.200702074) 17846176 10.1083/jcb.200702074 PMC2064629

[31] Liu M Nadar VC Kozielski F Kozlowska M Yu W Baas PW 2010 Kinesin-12, a mitotic microtubule-associated motor protein, impacts axonal growth, navigation, and branching. J. Neurosci. 30, 14 896–14 906 (doi:10.1523/jneurosci.3739-10.2010) 10.1523/JNEUROSCI.3739-10.2010 PMC3064264 21048148

[32] Falnikar A Tole S Baas PW 2011 Kinesin-5, a mitotic microtubule-associated motor protein, modulates neuronal migration. Mol. Biol. Cell 22, 1561–1574 (doi:10.1091/mbc.E10-11-0905) 21411631 10.1091/mbc.E10-11-0905 PMC3084678

[33] Lin S Liu M Mozgova OI Yu W Baas PW 2012 Mitotic motors coregulate microtubule patterns in axons and dendrites. J. Neurosci. 32, 14 033–14 049 (doi:10.1523/jneurosci.3070-12.2012) 10.1523/JNEUROSCI.3070-12.2012 PMC3482493 23035110

[34] Gardner MK Zanic M Gell C Bormuth V Howard J 2011 Depolymerizing kinesins Kip3 and MCAK shape cellular microtubule architecture by differential control of catastrophe. Cell 147, 1092–1103 (doi:10.1016/j.cell.2011.10.037) 22118464 10.1016/j.cell.2011.10.037

[35] Gardner MK Zanic M Howard J 2013 Microtubule catastrophe and rescue. Curr. Opin. Cell Biol. 25, 14–22 (doi:10.1016/j.ceb.2012.09.006) 23092753 10.1016/j.ceb.2012.09.006 PMC3556214

[36] Gupta A Tsai LH Wynshaw-Boris A 2002 Life is a journey: a genetic look at neocortical development. Nat. Rev. Genet. 3, 342–355 (doi:10.1038/nrg799) 11988760 10.1038/nrg799

[37] Jaglin XH Chelly J 2009 Tubulin-related cortical dysgenesis: microtubule dysfunction underlying neuronal migration defects. Trends Genet. 25, 555–566 (doi:10.1016/j.tig.2009.10.003) 19864038 10.1016/j.tig.2009.10.003

[38] Wynshaw-Boris A Pramparo T Youn YH Hirotsune S 2010 Lissencephaly: mechanistic insights from animal models and potential therapeutic strategies. Semin. Cell Dev. Biol. 21, 823–830 (doi:10.1016/j.semcdb.2010.07.008) 20688183 10.1016/j.semcdb.2010.07.008 PMC2967611

[39] Manzini MC Walsh CA 2011 What disorders of cortical development tell us about the cortex: one plus one does not always make two. Curr. Opin. Genet. Dev. 21, 333–339 (doi:10.1016/j.gde.2011.01.006) 21288712 10.1016/j.gde.2011.01.006 PMC3139684

[40] Tischfield MA Cederquist GY Gupta ML Jr Engle EC 2011 Phenotypic spectrum of the tubulin-related disorders and functional implications of disease-causing mutations. Curr. Opin. Genet. Dev. 21, 286–294 (doi:10.1016/j.gde.2011.01.003) 21292473 10.1016/j.gde.2011.01.003 PMC3100401

[41] Reiner O 2013 LIS1 and DCX: implications for brain development and human disease in relation to microtubules. Scientifica 2013, 393975 (doi:10.1155/2013/393975) 10.1155/2013/393975 PMC3820303 24278775

[42] Keays DA 2007 Mutations in α-tubulin cause abnormal neuronal migration in mice and lissencephaly in humans. Cell 128, 45–57 (doi:10.1016/j.cell.2006.12.017) 17218254 10.1016/j.cell.2006.12.017 PMC1885944

[43] Jaglin XH 2009 Mutations in β-tubulin gene *TUBB2B* result in asymmetrical polymicrogyria. Nat. Genet. 41, 746–752 (doi:10.1038/ng.380) 19465910 10.1038/ng.380 PMC2883584

[44] Tischfield MA 2010 Human *TUBB3* mutations perturb microtubule dynamics, kinesin interactions, and axon guidance. Cell 140, 74–87 (doi:10.1016/j.cell.2009.12.011) 20074521 10.1016/j.cell.2009.12.011 PMC3164117

[45] Smith DS Niethammer M Ayala R Zhou Y Gambello MJ Wynshaw-Boris A Tsai LH 2000 Regulation of cytoplasmic dynein behaviour and microtubule organization by mammalian Lis1. Nat. Cell Biol. 2, 767–775 (doi:10.1038/35041000) 11056530 10.1038/35041000

[46] Faulkner NE Dujardin DL Tai CY Vaughan KT O'Connell CB Wang YI Vallee RB 2000 A role for the lissencephaly gene *LIS1* in mitosis and cytoplasmic dynein function. Nat. Cell Biol. 2, 784–791 (doi:10.1038/35041020) 11056532 10.1038/35041020

[47] Sasaki S Shionoya A Ishida M Gambello MJ Yingling J Wynshaw-Boris A Hirotsune S 2000 A LIS1/NUDEL/cytoplasmic dynein heavy chain complex in the developing and adult nervous system. Neuron 28, 681–696 (doi:10.1016/S0896-6273(00)00146-X) 11163259 10.1016/s0896-6273(00)00146-x

[48] Kerjan G Gleeson JG 2007 Genetic mechanisms underlying abnormal neuronal migration in classical lissencephaly. Trends Genet. 23, 623–630 (doi:10.1016/j.tig.2007.09.003) 17997185 10.1016/j.tig.2007.09.003

[49] Reiner O Carrozzo R Shen Y Wehnert M Faustinella F Dobyns WB Caskey CT Ledbetter DH 1993 Isolation of a Miller–Dieker lissencephaly gene containing G protein beta-subunit-like repeats. Nature 364, 717–721 (doi:10.1038/364717a0) 8355785 10.1038/364717a0

[50] Bi W 2009 Increased LIS1 expression affects human and mouse brain development. Nat. Genet. 41, 168–177 (doi:10.1038/ng.302) 19136950 10.1038/ng.302 PMC4396744

[51] des Portes V 1998 A novel CNS gene required for neuronal migration and involved in X-linked subcortical laminar heterotopia and lissencephaly syndrome. Cell 92, 51–61 (doi:10.1016/S0092-8674(00)80898-3) 9489699 10.1016/s0092-8674(00)80898-3

[52] Gleeson JG 1998 Doublecortin, a brain-specific gene mutated in human X-linked lissencephaly and double cortex syndrome, encodes a putative signaling protein. Cell 92, 63–72 (doi:10.1016/S0092-8674(00)80899-5) 9489700 10.1016/s0092-8674(00)80899-5

[53] Francis F 1999 Doublecortin is a developmentally regulated, microtubule-associated protein expressed in migrating and differentiating neurons. Neuron 23, 247–256 (doi:10.1016/S0896-6273(00)80777-1) 10399932 10.1016/s0896-6273(00)80777-1

[54] Gleeson JG Lin PT Flanagan LA Walsh CA 1999 Doublecortin is a microtubule-associated protein and is expressed widely by migrating neurons. Neuron 23, 257–271 (doi:10.1016/S0896-6273(00)80778-3) 10399933 10.1016/s0896-6273(00)80778-3

[55] Horesh D Sapir T Francis F Wolf SG Caspi M Elbaum M Chelly J Reiner O 1999 Doublecortin: a stabilizer of microtubules. Hum. Mol. Genet. 8, 1599–1610 (doi:10.1093/hmg/8.9.1599) 10441322 10.1093/hmg/8.9.1599

[56] Sapir T Frotscher M Levy T Mandelkow EM Reiner O 2011 Tau's role in the developing brain: implications for intellectual disability. Hum. Mol. Genet. 21, 1681–1692 (doi:10.1093/hmg/ddr603) 22194194 10.1093/hmg/ddr603

[57] Niblock M Gallo JM 2012 Tau alternative splicing in familial and sporadic tauopathies. Biochem. Soc. Trans. 40, 677–680 (doi:10.1042/bst20120091) 22817715 10.1042/BST20120091

[58] Tanaka T Serneo FF Higgins C Gambello MJ Wynshaw-Boris A Gleeson JG 2004 Lis1 and doublecortin function with dynein to mediate coupling of the nucleus to the centrosome in neuronal migration. J. Cell Biol. 165, 709–721 (doi:10.1083/jcb.200309025) 15173193 10.1083/jcb.200309025 PMC2172383

[59] Tsai JW Bremner KH Vallee RB 2007 Dual subcellular roles for LIS1 and dynein in radial neuronal migration in live brain tissue. Nat. Neurosci. 10, 970–979 (doi:10.1038/nn1934) 17618279 10.1038/nn1934

[60] Asada N Sanada K Fukada Y 2007 LKB1 regulates neuronal migration and neuronal differentiation in the developing neocortex through centrosomal positioning. J. Neurosci. 27, 11 769–11 775 (doi:10.1523/jneurosci.1938-07.2007) 10.1523/JNEUROSCI.1938-07.2007 PMC6673218 17959818

[61] Asada N Sanada K 2010 LKB1-mediated spatial control of GSK3β and adenomatous polyposis coli contributes to centrosomal forward movement and neuronal migration in the developing neocortex. J. Neurosci. 30, 8852–8865 (doi:10.1523/jneurosci.6140-09.2010) 20592207 10.1523/JNEUROSCI.6140-09.2010 PMC6632896

[62] Sapir T Sapoznik S Levy T Finkelshtein D Shmueli A Timm T Mandelkow EM Reiner O 2008 Accurate balance of the polarity kinase MARK2/Par-1 is required for proper cortical neuronal migration. J. Neurosci. 28, 5710–5720 (doi:10.1523/jneurosci.0911-08.2008) 18509032 10.1523/JNEUROSCI.0911-08.2008 PMC6670809

[63] Sapir T Shmueli A Levy T Timm T Elbaum M Mandelkow EM Reiner O 2008 Antagonistic effects of doublecortin and MARK2/Par-1 in the developing cerebral cortex. J. Neurosci. 28, 13 008–13 013 (doi:10.1523/jneurosci.2363-08.2008) 10.1523/JNEUROSCI.2363-08.2008 PMC6671805 19036994

[64] Rivas RJ Hatten ME 1995 Motility and cytoskeletal organization of migrating cerebellar granule neurons. J. Neurosci. 15, 981–989 7869123 10.1523/JNEUROSCI.15-02-00981.1995 PMC6577828

[65] Umeshima H Hirano T Kengaku M 2007 Microtubule-based nuclear movement occurs independently of centrosome positioning in migrating neurons. Proc. Natl Acad. Sci. USA 104, 16 182–16 187 (doi:10.1073/pnas.0708047104) 10.1073/pnas.0708047104 PMC2000450 17913873

[66] Distel M Hocking JC Volkmann K Köster RW 2010 The centrosome neither persistently leads migration nor determines the site of axonogenesis in migrating neurons *in vivo*. J. Cell Biol. 191, 875–890 (doi:10.1083/jcb.201004154) 21059852 10.1083/jcb.201004154 PMC2983064

[67] Sakakibara A Sato T Ando R Noguchi N Masaoka M Miyata T In press Dynamics of centrosome translocation and microtubule organization in neocortical neurons during distinct modes of polarization. Cereb. Cortex. (doi:10.1093/cercor/bhs411) 10.1093/cercor/bhs411 23307632

[68] Solecki DJ Trivedi N Govek EE Kerekes RA Gleason SS Hatten ME 2009 Myosin II motors and F-actin dynamics drive the coordinated movement of the centrosome and soma during CNS glial-guided neuronal migration. Neuron 63, 63–80 (doi:10.1016/j.neuron.2009.05.028) 19607793 10.1016/j.neuron.2009.05.028 PMC2737100

[69] Bellion A Baudoin JP Alvarez C Bornens M Metin C 2005 Nucleokinesis in tangentially migrating neurons comprises two alternating phases: forward migration of the Golgi/centrosome associated with centrosome splitting and myosin contraction at the rear. J. Neurosci. 25, 5691–5699 (doi:10.1523/jneurosci.1030-05.2005) 15958735 10.1523/JNEUROSCI.1030-05.2005 PMC6724882

[70] Schaar BT McConnell SK 2005 Cytoskeletal coordination during neuronal migration. Proc. Natl Acad. Sci. USA 102, 13 652–13 657 (doi:10.1073/pnas.0506008102) 10.1073/pnas.0506008102 PMC1199551 16174753

[71] Martini FJ Valdeolmillos M 2010 Actomyosin contraction at the cell rear drives nuclear translocation in migrating cortical interneurons. J. Neurosci. 30, 8660–8670 (doi:10.1523/jneurosci.1962-10.2010) 20573911 10.1523/JNEUROSCI.1962-10.2010 PMC6634617

[72] Dotti CG Sullivan CA Banker GA 1988 The establishment of polarity by hippocampal neurons in culture. J. Neurosci. 8, 1454–1468 3282038 10.1523/JNEUROSCI.08-04-01454.1988 PMC6569279

[73] Cáceres A Ye B Dotti CG 2012 Neuronal polarity: demarcation, growth and commitment. Curr. Opin. Cell Biol. 24, 547–553 (doi:10.1016/j.ceb.2012.05.011) 22726583 10.1016/j.ceb.2012.05.011 PMC3425660

[74] Hatanaka Y Yamauchi K Murakami F 2012 Formation of axon-dendrite polarity *in situ*: initiation of axons from polarized and non-polarized cells. Dev. Growth Differ. 54, 398–407 (doi:10.1111/j.1440-169X.2012.01344.x) 22524609 10.1111/j.1440-169X.2012.01344.x

[75] Shi SH Jan LY Jan YN 2003 Hippocampal neuronal polarity specified by spatially localized mPar3/mPar6 and PI 3-kinase activity. Cell 112, 63–75 (doi:10.1016/S0092-8674(02)01249-7) 12526794 10.1016/s0092-8674(02)01249-7

[76] Ménager C Arimura N Fukata Y Kaibuchi K 2004 PIP_3_ is involved in neuronal polarization and axon formation. J. Neurochem. 89, 109–118 (doi:10.1046/j.1471-4159.2004.02302.x) 15030394 10.1046/j.1471-4159.2004.02302.x

[77] Schwamborn JC Püschel AW 2004 The sequential activity of the GTPases Rap1B and Cdc42 determines neuronal polarity. Nat. Neurosci. 7, 923–929 (doi:10.1038/nn1295) 15286792 10.1038/nn1295

[78] Fukata Y 2002 CRMP-2 binds to tubulin heterodimers to promote microtubule assembly. Nat. Cell Biol. 4, 583–591 (doi:10.1038/ncb825) 12134159 10.1038/ncb825

[79] Yoshimura T Kawano Y Arimura N Kawabata S Kikuchi A Kaibuchi K 2005 GSK-3β regulates phosphorylation of CRMP-2 and neuronal polarity. Cell 120, 137–149 (doi:10.1016/j.cell.2004.11.012) 15652488 10.1016/j.cell.2004.11.012

[80] Horiguchi K Hanada T Fukui Y Chishti AH 2006 Transport of PIP_3_ by GAKIN, a kinesin-3 family protein, regulates neuronal cell polarity. J. Cell Biol. 174, 425–436 (doi:10.1083/jcb.200604031) 16864656 10.1083/jcb.200604031 PMC2064238

[81] Gdalyahu A Ghosh I Levy T Sapir T Sapoznik S Fishler Y Azoulai D Reiner O 2004 DCX, a new mediator of the JNK pathway. EMBO J. 23, 823–832 (doi:10.1038/sj.emboj.7600079) 14765123 10.1038/sj.emboj.7600079 PMC380994

[82] Kishi M Pan YA Crump JG Sanes JR 2005 Mammalian SAD kinases are required for neuronal polarization. Science 30, 929–932 (doi:10.1126/science.1107403) 15705853 10.1126/science.1107403

[83] Barnes AP Lilley BN Pan YA Plummer LJ Powell AW Raines AN Sanes JR Polleux F 2007 LKB1 and SAD kinases define a pathway required for the polarization of cortical neurons. Cell 129, 549–563 (doi:10.1016/j.cell.2007.03.025) 17482548 10.1016/j.cell.2007.03.025

[84] Shelly M Cancedda L Heilshorn S Sumbre G Poo MM 2007 LKB1/STRAD promotes axon initiation during neuronal polarization. Cell 129, 565–577 (doi:10.1016/j.cell.2007.04.012) 17482549 10.1016/j.cell.2007.04.012

[85] Hirai S Banba Y Satake T Ohno S 2011 Axon formation in neocortical neurons depends on stage-specific regulation of microtubule stability by the dual leucine zipper kinase-c-Jun N-terminal kinase pathway. J. Neurosci. 31, 6468–6480 (doi:10.1523/jneurosci.5038-10.2011) 21525288 10.1523/JNEUROSCI.5038-10.2011 PMC6622676

[86] Witte H Neukirchen D Bradke F 2008 Microtubule stabilization specifies initial neuronal polarization. J. Cell Biol. 180, 619–632 (doi:10.1083/jcb.200707042) 18268107 10.1083/jcb.200707042 PMC2234250

[87] Schaar BT Kinoshita K McConnell SK 2004 Doublecortin microtubule affinity is regulated by a balance of kinase and phosphatase activity at the leading edge of migrating neurons. Neuron 41, 203–213 (doi:10.1016/S0896-6273(03)00843-2) 14741102 10.1016/s0896-6273(03)00843-2

[88] Chen YM Wang QJ Hu HS Yu PC Zhu J Drewes G Piwnica-Worms H Luo ZG 2006 Microtubule affinity-regulating kinase 2 functions downstream of the PAR-3/PAR-6/atypical PKC complex in regulating hippocampal neuronal polarity. Proc. Natl Acad. Sci. USA 103, 8534–8539 (doi:10.1073/pnas.0509955103) 16717194 10.1073/pnas.0509955103 PMC1482526

[89] Eto K Kawauchi T Osawa M Tabata H Nakajima K 2010 Role of dual leucine zipper-bearing kinase (DLK/MUK/ZPK) in axonal growth. Neurosci. Res. 66, 37–45 (doi:10.1016/j.neures.2009.09.1708) 19808064 10.1016/j.neures.2009.09.1708

[90] Westerlund N 2011 Phosphorylation of SCG10/stathmin-2 determines multipolar stage exit and neuronal migration rate. Nat. Neurosci. 14, 305–313 (doi:10.1038/nn.2755) 21297631 10.1038/nn.2755

[91] Watabe-Uchida M John KA Janas JA Newey SE Van Aelst L 2006 The Rac activator DOCK7 regulates neuronal polarity through local phosphorylation of stathmin/Op18. Neuron 51, 727–739 (doi:10.1016/j.neuron.2006.07.020) 16982419 10.1016/j.neuron.2006.07.020

[92] Lefcort F Bentley D 1989 Organization of cytoskeletal elements and organelles preceding growth cone emergence from an identified neuron *in situ*. J. Cell Biol. 108, 1737–1749 (doi:10.1083/jcb.108.5.1737) 2654140 10.1083/jcb.108.5.1737 PMC2115544

[93] Zmuda JF Rivas RJ 1998 The Golgi apparatus and the centrosome are localized to the sites of newly emerging axons in cerebellar granule neurons *in vitro*. Cell Motil. Cytoskeleton 41, 18–38 (doi:10.1002/(SICI)1097-0169(1998)41:1<18::AID-CM2>3.0.CO;2-B) 9744296 10.1002/(SICI)1097-0169(1998)41:1<18::AID-CM2>3.0.CO;2-B

[94] de Anda FC Pollarolo G Da Silva JS Camoletto PG Feiguin F Dotti CG 2005 Centrosome localization determines neuronal polarity. Nature 436, 704–708 (doi:10.1038/nature03811) 16079847 10.1038/nature03811

[95] de Anda FC Meletis K Ge X Rei D Tsai L-H 2010 Centrosome motility is essential for initial axon formation in the neocortex. J. Neurosci. 30, 10 391–10 406 (doi:10.1523/jneurosci.0381-10.2010) 10.1523/JNEUROSCI.0381-10.2010 PMC6634663 20685982

[96] Gärtner A Fornasiero EF Munck S Vennekens K Seuntjens E Huttner WB Valtorta F Dotti CG 2012 N-cadherin specifies first asymmetry in developing neurons. EMBO J. 31, 1893–1903 (doi:10.1038/emboj.2012.41) 22354041 10.1038/emboj.2012.41 PMC3343329

[97] Zolessi FR Poggi L Wilkinson CJ Chien CB Harris WA 2006 Polarization and orientation of retinal ganglion cells *in vivo*. Neural Dev. 1, 2 (doi:10.1186/1749-8104-1-2) 17147778 10.1186/1749-8104-1-2 PMC1636330

[98] Burton PR Paige JL 1981 Polarity of axoplasmic microtubules in the olfactory nerve of the frog. Proc. Natl Acad. Sci. USA 78, 3269–3273 (doi:10.1073/pnas.78.5.3269) 6973153 10.1073/pnas.78.5.3269 PMC319543

[99] Heidemann SR Landers JM Hamborg MA 1981 Polarity orientation of axonal microtubules. J. Cell Biol. 91, 661–665 (doi:10.1083/jcb.91.3.661) 6173385 10.1083/jcb.91.3.661 PMC2112798

[100] Baas PW Deitch JS Black MM Banker GA 1988 Polarity orientation of microtubules in hippocampal neurons: uniformity in the axon and nonuniformity in the dendrite. Proc. Natl Acad. Sci. USA 85, 8335–8339 (doi:10.1073/pnas.85.21.8335) 3054884 10.1073/pnas.85.21.8335 PMC282424

[101] Baas PW Black MM Banker GA 1989 Changes in microtubule polarity orientation during the development of hippocampal neurons in culture. J. Cell Biol. 109, 3085–3094 (doi:10.1083/jcb.109.6.3085) 2592416 10.1083/jcb.109.6.3085 PMC2115969

[102] Rakic P Knyihar-Csillik E Csillik B 1996 Polarity of microtubule assemblies during neuronal cell migration. Proc. Natl Acad. Sci. USA 93, 9218–9222 (doi:10.1073/pnas.93.17.9218) 8799181 10.1073/pnas.93.17.9218 PMC38622

[103] Stepanova T 2003 Visualization of microtubule growth in cultured neurons via the use of EB3-GFP (end-binding protein 3-green fluorescent protein). J. Neurosci. 23, 2655–2664 12684451 10.1523/JNEUROSCI.23-07-02655.2003 PMC6742099

[104] Rolls MM Satoh D Clyne PJ Henner AL Uemura T Doe CQ 2007 Polarity and intracellular compartmentalization of *Drosophila* neurons. Neural Dev. 2, 7 (doi:10.1186/1749-8104-2-7) 17470283 10.1186/1749-8104-2-7 PMC1868948

[105] Maniar TA Kaplan M Wang GJ Shen K Wei L Shaw JE Koushika SP Bargmann CI 2012 UNC-33 (CRMP) and ankyrin organize microtubules and localize kinesin to polarize axon-dendrite sorting. Nat. Neurosci. 15, 48–56 (doi:10.1038/nn.2970) 22101643 10.1038/nn.2970 PMC4328884

[106] Goodwin PR Sasaki JM Juo P 2012 Cyclin-dependent kinase 5 regulates the polarized trafficking of neuropeptide-containing dense-core vesicles in *Caenorhabditis elegans* motor neurons. J. Neurosci. 32, 8158–8172 (doi:10.1523/jneurosci.0251-12.2012) 22699897 10.1523/JNEUROSCI.0251-12.2012 PMC3392131

[107] Zheng Y Wildonger J Ye B Zhang Y Kita A Younger SH Zimmerman S Jan LY Jan YN 2008 Dynein is required for polarized dendritic transport and uniform microtubule orientation in axons. Nat. Cell Biol. 10, 1172–1180 (doi:10.1038/ncb1777) 18758451 10.1038/ncb1777 PMC2588425

[108] Satoh D Sato D Tsuyama T Saito M Ohkura H Rolls MM Ishikawa F Uemura T 2008 Spatial control of branching within dendritic arbors by dynein-dependent transport of Rab5-endosomes. Nat. Cell Biol. 10, 1164–1171 (doi:10.1038/ncb1776) 18758452 10.1038/ncb1776

[109] Ori-McKenney KM Jan LY Jan YN 2012 Golgi outposts shape dendrite morphology by functioning as sites of acentrosomal microtubule nucleation in neurons. Neuron 76, 921–930 (doi:10.1016/j.neuron.2012.10.008) 23217741 10.1016/j.neuron.2012.10.008 PMC3523279

[110] Horton AC Racz B Monson EE Lin AL Weinberg RJ Ehlers MD 2005 Polarized secretory trafficking directs cargo for asymmetric dendrite growth and morphogenesis. Neuron 48, 757–771 (doi:10.1016/j.neuron.2005.11.005) 16337914 10.1016/j.neuron.2005.11.005

[111] Roll-Mecak A Vale RD 2006 Making more microtubules by severing: a common theme of noncentrosomal microtubule arrays? J. Cell Biol. 175, 849–851 (doi:10.1083/jcb.200611149) 17178905 10.1083/jcb.200611149 PMC2064694

[112] Basto R Lau J Vinogradova T Gardiol A Woods CG Khodjakov A Raff JW 2006 Flies without centrioles. Cell 125, 1375–1386 (doi:10.1016/j.cell.2006.05.025) 16814722 10.1016/j.cell.2006.05.025

[113] Nguyen MM Stone MC Rolls MM 2011 Microtubules are organized independently of the centrosome in *Drosophila* neurons. Neural Dev. 6, 38 (doi:10.1186/1749-8104-6-38) 22145670 10.1186/1749-8104-6-38 PMC3271965

[114] Stiess M Maghelli N Kapitein LC Gomis-Rüth S Wilsch-Bräuninger M Hoogenraad CC Tolić-Nørrelykke IM Bradke F 2010 Axon extension occurs independently of centrosomal microtubule nucleation. Science 327, 704–707 (doi:10.1126/science.1182179) 20056854 10.1126/science.1182179

[115] Yu W Centonze VE Ahmad FJ Baas PW 1993 Microtubule nucleation and release from the neuronal centrosome. J. Cell Biol. 122, 349–359 (doi:10.1083/jcb.122.2.349) 8320258 10.1083/jcb.122.2.349 PMC2119640

